# Individual Differences in Tendencies Toward Internet Use Disorder, Internet Literacy and Their Link to Autistic Traits in Both China and Germany

**DOI:** 10.3389/fpsyt.2021.638655

**Published:** 2021-09-27

**Authors:** YingYing Zhang, Cornelia Sindermann, Keith M. Kendrick, Benjamin Becker, Christian Montag

**Affiliations:** ^1^Department of Molecular Psychology, Institute of Psychology and Education, Ulm University, Ulm, Germany; ^2^Ministry of Education Key Lab for Neuroinformation, The Clinical Hospital of Chengdu Brain Science Institute, University of Electronic Science and Technology of China, Chengdu, China

**Keywords:** internet use disorder, internet addiction, autistic traits, internet literacy, China, Germany

## Abstract

Recent evidence demonstrates that Internet Use Disorder tendencies (IUD; formerly known as Internet Addiction) are associated with higher tendencies toward autistic traits. In the present study, we aimed to further explore this association between IUD tendencies and autistic traits in a large cohort of German and Chinese subjects (total *N* = 1,524; mostly student background) who completed the short Internet-Addiction-Test, the Autism-Spectrum-Quotient, and the Internet-Literacy-Questionnaire. Moreover, the present research also enabled us to study potential differences in the investigated variables between the Chinese and German cultures. First, the results indicated higher occurrence of IUD symptoms in China. Moreover, Chinese subjects scored significantly higher on all ILQ dimensions than German participants, with the exception of *self-regulation* where the reverse picture appeared. Second, results confirmed a positive association between IUD tendencies and autistic traits both in China and Germany, although effect sizes were low to medium (China: *r* = 0.19 vs. Germany: *r* = 0.36). Going beyond the literature, the present study also assessed individual differences in Internet Literacy and shows in how far variables such as *technical expertise, production and interaction, reflection and critical analysis* as well as *self-regulation* in the realm of the Internet usage influence the aforementioned association between IUD tendencies and autistic traits. Although the present study is limited by being of correlational nature it is discussed how the association between IUD tendencies and autistic traits might be explained.

## Introduction

Currently, 65.6% of the world's population have access to the Internet^1^. The rapid increase in available digital technologies has brought many advantages such as access to useful information, entertainment and the possibility of exchanging texts, pictures and video messages almost anywhere at any time. In particular, it has made long-distance communication far easier and cheaper. However, in addition to these positive aspects there is increasing concern and on-going debate over whether over-usage of online channels may represent a threat to mental health (1). Indeed, excessive usage of the Internet has been associated with attention deficit hyperactivity disorder (ADHD), depression, and social phobia (2–4) as well with autistic traits [e.g., (5)]. However, whether over-usage of the Internet contributes causally to these disorders is largely unresolved.

Since the initial report of a female patient potentially being “addicted” to the online world (6), a rapidly growing body of evidence demonstrates that excessive use of the Internet could indeed represent a mental health problem [for a recent overview see the work by (7)]. In this context it is of importance to mention that the term “Internet Addiction” is heavily criticized and most researchers in the field currently prefer to use the term problematic Internet use or, in line with recent developments in ICD-11, Internet Use Disorder [IUD; e.g., (8)]: In 2013 the American Psychiatric Association (APA) added Internet Gaming Disorder as an emerging disorder in section 3 of DSM-5's appendix (9). Here, Internet Gaming Disorder as a specific form of IUD was recognized for the first time as a potential mental health problem by an official health organization. The inclusion of this scientific working term triggered more structured research in the field, with the outcome that the World Health Organization (WHO) has now even included Gaming Disorder as an officially recognized disorder in ICD-11^2^. In line with this terminology the I-PACE model by Brand et al. (10, 11) perhaps currently represents the most comprehensive model for understanding and studying IUD. The model proposes that an interaction of person, affect, cognition and execution variables can explain how individuals develop from habitual to pathological online use and it also draws on an older model by Davis (12) which makes the important distinction between unspecified (generalized) and specific IUDs. Whereas, unspecified IUD (also at the heart of this work) might function as an umbrella term describing individuals being hooked to several online channels such as online gaming and online social media, specific IUD explicitly refers to over-usage of only one online channel. The importance of distinguishing between specific and unspecific/unspecified IUDs has also been shown empirically by correlating different forms of online addictive behaviors with each other [showing only in parts high overlap (13, 14)]. In the present study, we mainly want to explore the development of unspecific IUD. Although no consensus has been reached on how to best diagnose and assess IUD, many researchers rely on measures based on an addiction background and also the compulsive-obsessive spectrum (15, 16). This all said, the term IUD itself is not officially recognized by the WHO or the APA. We explicitly mention that we use this term in the present manuscript to aim at unification of terms in the literature (in line with the aforementioned official Gaming Disorder diagnosis as a guiding light). In the realm of this discussion, it is of importance to mention that we do not want to over-pathologize everyday life behavior (17) and we also mention a recent paper by Elhai et al. (18) advocating for fairness principles in labeling Internet Use Disorders. In the present study, we mainly want to explore variables of potential interest for the development and maintenance of unspecific IUD.

Interestingly the prevalence rates of unspecific IUD are much higher in Asian countries (19), in particular higher numbers have been reported for Taiwan [around 13.8% (20)] and South Korea (around 10.7%) compared to lower numbers in European countries [around 4.4% (21, 22)]. Although clearly differences in prevalence rates across countries could be influenced by cultural background, they might also result from different measures used to assess IUD in previous studies and their respective sample characteristics. Cultural differences can be hard to detect due to the importance of samples from different countries being recruited in parallel and with matched socio-demographic variables such as age, gender and education. Nevertheless, Montag (23) has proposed that cross-cultural research can actually be an effective solution for resolving the replication crisis in psychology and the life sciences: if the same results can be observed across samples from different cultural backgrounds (especially, when the samples are also different in terms of gender and/or age, etc.), this provides compelling support for globally valid effects.

Although much research has been conducted on IUD tendencies in the last 20 years, only a few studies have attempted to link autistic traits with excessive online usage. Such a link is imaginable, because aside from links to ADHD and depression, IUD has been associated with both lower empathy and higher social anxiety (24–27). The latter psychological constructs are clearly known to play a relevant role to understand the autistic condition (28–30). Given the links between low empathy/high social anxiety and higher autistic traits—and against the background of the mentioned research—it seems likely that IUD and autistic traits could be related. Indeed, Finkenauer et al. (31) reported that strong autistic traits could predict compulsive Internet use in adults. Romano et al. (5) further found that higher levels of IUD were associated with higher levels of autistic traits and this association was particularly pronounced in participants with higher anxiety levels. Given the scarce literature on this topic, we revisited with the present research the question on the relationship between autistic traits and IUD tendencies. Please note that we investigated autistic traits in subclinical samples in the present work, but we are also referring to recent clinical work showing either elevated compulsive Internet use in patients with Autism Spectrum Disorder (ASD) rated by parents (32) or high prevalence of IUD in patients with ASD [10.8% (33)]. Of further interest, the existing studies investigating links between the autistic traits and IUD tendencies did not investigate how facets of the autistic traits are linked to individual differences in tendencies toward IUD. The questionnaire measuring autistic traits (the Autism Spectrum Questionnaire; AQ) can be split into facets called *social skills, attention switching, attention to details, communication* and *imagination* (34). Against the background of studying individual differences in IUD tendencies, we believe the AQ facets *social skills* and *communication* to be of particular importance. Individuals scoring high on AQ's *social skills* among others prefer to do things alone and do not like social situations. Individuals scoring high on the AQ's *communication* among others describe themselves as being recognized as impolite by others and having difficulties to keep a talk going. Although speculative, one could expect that in particular problems in *social skill/communication* areas might go along with higher IUD tendencies, because the Internet might be seen by persons with higher autistic traits as enabling communication without being directly confronted with others. This might be in ways good (as a first solution to enable communication). But when habits are formed to only live one's own life via the Internet, social withdrawal together with IUD patterns might result in pronounced negative affect.

Next to investigating the associations between IUD and autistic tendencies including various facets of autistic tendencies and going beyond the already published works in the field, we aimed to understand whether the association between IUD tendencies and autistic traits would be illuminated by different dimensions of Internet Literacy. In older work by Stodt et al. (35), it was already demonstrated that the facets of Internet Literacy *technical expertise* and *knowledge about production and interaction* in the context of Internet use are positively associated with IUD tendencies, whereas the facet of Internet Literacy called *self-regulation* is negatively associated with IUD tendencies both in China and Germany. The present work re-examines this question in new, larger samples from China and Germany, and additionally takes into account the variable of autistic traits. We hypothesized that the aforementioned variables from Internet Literacy might influence the association between IUD tendencies and autistic traits. E.g., such an association might be stronger in individuals scoring lower on *self-regulation* and higher on *production and interaction* (which might be related to systemizing tendencies in autistic traits). In particular, Internet Literacy's *production and interaction* could be a moderator variable between autistic traits and IUD tendencies, because high scorers on this facet prefer online-over offline-communication, which is something very likely to occur in individuals scoring high on autistic traits and being drawn to overuse the Internet. Beyond this, variables related to Internet Literacy could be of special importance in the study of autistic traits and IUD tendencies, because according to a work by Stodt et al. (36) teaching self-regulation skills might help Internet users to reduce problems with overuse of the Internet, although this study did not deal with autistic traits. The ILQ part of the present work is of an exploratory nature and correction procedures for multiple testing will be applied to lower the chance for false positive findings. We also revisit the finding from Romano et al. (5) reporting that the association between AQ and IUD tendencies is particularly pronounced in individuals characterized by high trait anxiety. These findings are presented in the Supplementary Material (part 2). In summary, the main rationale for investigating autistic traits in the context of IUD might be grounded in the idea that the Internet facilitates communication for individuals with higher autistic traits, as the Internet offers more ways to indirectly communicate without complex non-verbal cues (37). This in turn might in some cases result in higher tendencies toward IUD. We already provided some thoughts on possible associations between AQ's facets *social skills/communication* and IUD tendencies, but also mention the exploratory character of the present study, here.

## Methods

### Participants

We invited participants from China and Germany to take part in the online surveys. Since we mainly followed a dimensional approach in the present study investigating tendencies toward IUD and autistic traits (vs. “only” focusing on clinical diagnoses), we had no exclusion criteria at the recruiting stage with respect to mental health or other medical conditions. A total of 1,524 participants (865 males, 659 females) were included in our study (please note that before getting to this final sample size, most prominently *n* = 2 individuals in the German sample and *n* = 53 individuals from the Chinese sample were excluded due to careless responding (filling in same answers >80% of the variables regarding the AQ scores, where inverted items can be found). Further reasons to be excluded were minor age, participating in the study by Stodt et al. (35) and participating twice (in the context of participating twice one data set was eliminated). Please note that this rate of careless responding is lower than what has been reported elsewhere (38, 39), but in the context of various operationalizations of careless responding such comparisons need to be considered with caution. A subgroup of the here analyzed participants of the present samples from China/Germany has been investigated in the context of molecular genetics of autistic traits and experimental research (40, 41). Moreover, an overlap exists between the present sample and the sample reported in a recent work by Sindermann et al. (30) investigating autistic traits and empathy in a German sample.

The mean-age of the whole sample was M = 22.13 years old (SD = 5.46). The whole procedure was the same in China and Germany including protocols for questionnaires analyzed in our work and the online platform, which subjects used to fill in the questionnaires.

All Chinese participants were recruited as part of the Chengdu Gene Brain Behavior Project (CGBBP) and included 929 participants (sex: 696 males, 233 females; age: M = 21.48 years, SD = 2.29, range: 18–32). The majority of subjects were university students, in detail: 91.6% of the sample. The study was approved by the local ethics committee at the University of Electronic Science and Technology of China (UESTC). All subjects provided electronic informed consent and received monetary compensation for their participation.

The 595 German subjects recruited for the current study were recruited in course of the Ulm Gene Brain Behavior Project (UGBBP) (sex: 169 males, 426 females; age: M = 23.16, SD = 8.15, range: 18–82). Participants were recruited mainly from Ulm, Germany by advertisements and 86.6% of the participants were university students. Some questionnaire data was missing from 10 subjects in the German sample (<2%) and their missing data was handled by imputation from the mean (42). All participants provided electronic informed consent and received monetary compensation for their efforts. The German part of the study was approved by the local ethics' committee of Ulm University, Ulm, Germany.

### Self-Report Measures

The online platform SurveyCoder programmed by Christopher Kannen (ckannen.com) was used to collect subjects' self-report information via the administration of the below mentioned questionnaires. Although both the CGBBP and UGBBP included a large battery of self-report questionnaires to grasp individual differences in different traits, in the present work, we focused on the Adult Autism Spectrum Quotient (AQ) (mentioned above), the short Internet Addiction Test (s-IAT) and due to the work by Stodt et al. (35) on the Internet Literacy Questionnaire (ILQ). Chinese and German versions of all questionnaires were already available [e.g., as used in (35)]. In this study, all questionnaires and their subscales revealed a good internal consistency with all Cronbach's α > 0.700, except for AQ total scores in the Chinese sample, and several AQ subscales in both samples (Table 1).

**Table 1:** The reliabilities (Cronbach's α) of the short Internet Addiction Test (s-IAT), Internet Literacy Questionnaire (ILQ), and the Autism Spectrum Quotient (AQ) and their subscales in the Chinese and German samples.

**Domain/variable**	**China**	**Germany**	**Scale range^a^**
**Short Internet Addiction Test (s-IAT)**	0.885	0.851	12–60
sIAT Loss of control/time management	0.821	0.794	6–30
sIAT Craving/social problems	0.798	0.757	6–30
**Internet Literacy Questionnaire (ILQ)**			
ILQ Technical expertise	0.770	0.784	0–5
ILQ Production and interaction	0.811	0.817	0–5
ILQ Reflection and critical analysis	0.717	0.784	0–5
ILQ Self-regulation	0.754	0.783	0–5
**Autism Spectrum Questionnaire (AQ)**	0.674	0.725	0–50
AQ Social skills	0.711	0.709	0–10
AQ Attention switching	0.278	0.426	0–10
AQ Attention to details	0.478	0.513	0–10
AQ Communication	0.526	0.571	0–10
AQ Imagination	0.359	0.381	0–10

a *Scale range means the possible range of each questionnaire scale*.

*Reliabilities were calculated on complete data sets provided by the participants, hence, participants who provided missing data were excluded from the calculations of reliabilities of the scales*.

### Autism Spectrum Quotient (AQ)

Based on the hypothesis that autism is a dimensional rather than a categorial variable (i.e., can be measured in healthy as well as patient populations), Baron-Cohen et al. (34) developed the widely used AQ, which assessed individual differences in autistic traits in subclinical groups. It contains 50 items and all of the answers are based on a four-point Likert scale (from “definitely agree” to “definitely disagree;” with response options reduced to a scoring of 0/1 during data analysis as described in the original works). The AQ also includes items with reverse statements requiring recoding in the opposite way (from definitely disagree to definitely agree). The test-retest reliability of this questionnaire is 0.70, and the internal consistency has previously been found to be 0.82 (34). Internal consistencies for the present data are presented in Table 1 (please see that internal consistencies of most AQ subscales are in the low area of acceptability both in China and Germany).

With respect to the analysis of AQ scores presented in the results section, a quartile categorization method was used to divide participants into three different subgroups: low AQ scoring group (25% of participants below 25th quartile), medium AQ scoring group (50% of participants between 25th and 75th quartile) and high AQ scoring group (25% of participants above 75th quartile) in both countries. Participants with the same AQ scores were included in the same group and thus the groups do not exactly comprise the stated percentages of participants. It is worth noting that we mainly focused on the low and high scoring groups to explore associations with IUD tendencies and potential interactions with Internet Literacy. For reasons of completeness, we present results (means and standard deviations) contrasting s-IAT, AQ and ILQ variables according to clinical-/screening-cut-off-scores for the ASD (43) in the Supplementary Material.

### Short Internet Addiction Test (s-IAT)

As already mentioned, currently there is no consistent system for assessing IUD. We decided to choose the short Internet Addiction Test (s-IAT) developed by Pawlikowski et al. (44), which is a short version from Young's original IAT, which has shown excellent psychometric properties (44, 45). The s-IAT includes some of IUD's key elements such as loss of control and daily problems due to one's own excessive Internet usage. The s-IAT contains 12 items and each item is answered via a 5-point Likert scale from 1 (“never”) to 5 (“very often”). The questionnaire consists of the two subscales *loss of control/time management* and *craving/social problems*, each consisting of six items. The total scores of the s-IAT theoretically can vary between 12 and 60 points. Higher scores indicate higher problems due to one's own Internet use. According to Pawlikowski et al. (44) a score higher than 30 signifies problematic Internet use and higher than 37 signifies pathological Internet use.

### Internet Literacy Questionnaire (ILQ)

In order to assess individuals' competent and adequate dealing with the Internet use, we administered the Internet Literacy Questionnaire [ILQ; please find an early version of this questionnaire in Stodt et al. (36)]. According to a new exploratory factor analysis, a more economical version of the ILQ was used in this study [as presented in (35)]. The shortened version only includes 18 items of the original 24-item version, but also contains four dimensions as does the original version: *technical expertise, production and interaction, reflection and critical analysis*, and *self-regulation*. The dimension *technical expertise* measures “individuals' specialized knowledge in handling computer hard- and software as well as Internet applications” [(35), p. 31]; the dimension *production and interaction* explores “how and why an individual uses the Internet to create own content and to interact with others”. *Reflection and critical analysis* covers “individuals' ability to evaluate the credibility of online content and behavior of others as well as critically reflecting one's activities on the Internet” [(35), p. 31]. The last dimension *self-regulation* measures the ability to regulate individual's own Internet use to prevent negative consequences for daily life [(35), p. 31]. Each item is rated on a 6-point Likert scale from 0 (“strongly disagree”) to 5 (“totally agree”). Please note, that in the present work the mean scores are presented for each dimension. In our samples, the dimensions' internal consistencies (Cronbach's α) were satisfying for the German and Chinese versions (see Table 1).

### Data Analysis

First, we checked all distributions of s-IAT scores, ILQ dimension scales and AQ scores separately in China and Germany. According to (46), skewness and kurtosis values of |0–2| and |0–7|, respectively can be taken as demonstrating sufficient normality. After checking the distributions of all scales, all scales had a skewness and kurtosis < ±2, and due to our large sample size, we decided to use parametric tests (47). Histograms as well as information about skewness and kurtosis of distributions are presented in the Supplementary Material.

Statistical analyses were performed via the software package IBM SPSS24 (IBM, Armonk, NY, USA). The following procedures describe the overall analysis strategy with findings presented either in the following result section or the Supplementary Material.

Descriptive statistics were computed to describe means and standard deviations of all scales. We used *t*-tests for independent samples to examine differences between Chinese and German samples regarding the manifold Internet variables and AQ scores, and Hedge's g as an effect size. According to (48), the reported effect sizes for *t*-tests around 0.20 can be seen as small, around 0.50 as medium and around 0.80 as large. Aside from this we used Pearson's correlations to investigate associations between s-IAT scores, ILQ dimension's scores, and AQ scores in both countries and Fisher's z tests were used to assess differences between two correlations stemming from non-identical sample size (i.e., to compare correlations found in the Chinese and German samples). Moreover, gender differences in each sample and associations of all variables of interest with age were calculated, and we also ran detailed analysis for the AQ/s-IAT and AQ/ILQ correlations depending on AQ facets.

To determine the “influence” (it is a correlational study) of every variable on IUD tendencies, we conducted hierarchical regression models to predict IUD separately in China and Germany. In these two models, s-IAT total scores (not *z*-standardized) were implemented as dependent variable without focusing on its subscales. The independent variables—after being transferred into standardized z-scores—were entered in three blocks. In the first block, participants' social-demographics (e.g., age, gender) were entered. In the second block, the personality variable (AQ score) was entered to examine the influence of autistic traits after accounting for the demographic factor. In the third block, the four dimensions of the Internet Literacy questionnaire were entered as predictors. In addition, moderated regression analyses were used to extend the ILQ-s-IAT findings from Stodt et al. (35). In detail, we explored possible interaction effects between nationality and autistic traits on the one hand (not investigated in the Stodt et al. work) and between nationality and Internet Literacy on the other hand. Aside from this, we also tested whether the effects of the autistic traits on the s-IAT were moderated by Internet Literacy. Moreover, for a clearer understanding of the relationship between autistic traits and IUD tendencies, we compared the AQ total scores as well as AQ subscales' scores within different Internet user groups (e.g., non-problematic Internet users, problematic Internet users and pathological Internet users). Finally, we also considered and calculated two previously described cut-offs for the AQ: the “clinical” threshold of ≥32 and the “screening” cut-off of ≥26 (34) to investigate differences in s-IAT scores and ILQ dimensions between low autistic traits and high autistic traits.

## Results

### Descriptive Statistics and Differences Between the Chinese and German Samples

Table 2 shows the descriptive statistics of all questionnaires used (s-IAT, ILQ, and AQ) separately for the Chinese and German samples. The results revealed significant differences in s-IAT, ILQ, and AQ scores between the two samples. The scores on the s-IAT were significantly higher in the Chinese compared to the German sample (large effect size). For Internet Literacy, Chinese subjects showed higher scores in the dimensions of *technical expertise, production and interaction* and *reflection and critical analysis* compared to German participants, but lower scores in the dimension *self-regulation* (here with moderate to large effect sizes). Regarding the AQ scores, Chinese subjects again showed significantly higher scores overall than the German subjects.

**Table 2:** Means (M) and Standard Deviations (SD) plus *t*-tests investigating all psychometric measures depending on the variable German vs. Chinese sample.

**Domain/Variable**	**China (*n* = 929)**		**Germany (*n* = 595)**		***t***	***p***	**Hedge's g**
	**M**	**SD**	**M**	**SD**			
**Short Internet Addiction Test (s-IAT)**							
s-IAT Total scores	32.14	8.09	25.82	7.04	*t*_(1,388.39)_ = 16.12	<0.001	0.82
s-IAT LoC/TM	17.44	4.42	15.66	4.51	*t*_(1,522)_ = 7.59	<0.001	0.40
s-IAT LoC/TM	14.70	4.30	10.16	3.31	*t*_(1,471.94)_ = 23.23	<0.001	1.15
**Internet Literacy Questionnaire (ILQ)**							
ILQ TE	3.15	0.99	2.44	1.12	*t*_(1,146.66)_ = 12.58	<0.001	0.68
ILQ PI	3.08	0.97	1.85	1.09	*t*_(1,160.73)_ = 22.30	<0.001	1.21
ILQ RCA	2.99	0.86	2.84	0.94	*t*_(1,183.50)_ = 3.15	0.002	0.17
ILQ SR	2.82	0.86	3.31	0.92	*t*_(1,201.94)_ =-10.37	<0.001	0.55
**Autism Spectrum Quotient (AQ)**							
AQ Total scores	21.71	5.82	16.90	5.68	*t*_(1,522)_ = 15.89	<0.001	0.83
AQ: High scoring group (CH = 239/G = 126)	28.88	2.81	25.23	3.23	*t*_(363)_ = 11.20	<0.001	1.23
AQ: Middle scoring group (CH = 457/G = 337)	21.84	2.24	16.51	2.60	*t*_(657.38)_ = 30.26	<0.001	2.21
AQ: Low scoring group (CH = 233/G = 132)	14.10	2.53	9.95	1.79	*t*_(345.43)_ = 18.25	<0.001	1.81

*TE, technical expertise, PI, production and interaction; RCA, reflection and critical analysis; SR, self-regulation; LoC/TM, loss of control/time management; C/SP, craving/social problems; CH, China; G, Germany*.

### Occurrence of Autistic Traits

With the AQ-score quartile categorization method described in the method section, we divided our Chinese and German samples into different groups (high, average, and low scoring groups), although we primarily focused on the high and low AQ scoring groups. A total of 239 subjects in the Chinese sample and 126 subjects in the German sample were in the high AQ scoring group, and 233 subjects in the Chinese sample and 132 subjects in the German sample were in the low AQ scoring group.

### Occurrence of IUD

Applying the standard cut-off scores of the s-IAT according to Pawlikowski et al. (44) resulted in different prevalence rates in the two samples for the different IUD categories. The distributions of IUD groups differed across the Chinese and German samples [χ^2^ = 172.30, df = 2, *p* < 0.001]. Problematic use of the Internet was found in 34.3% of the Chinese sample compared to only 17.8% in the German sample. Pathological use of the Internet in the Chinese sample was found in 22.4%, whereas in the German sample it was 5.5%.

### Investigating Associations Between Age/Gender and the Variables of Interest in China and Germany Separately

In line with the different ranges of age in the two cohorts (China, range 18–32; Germany, range 18–82), age differed significantly between two samples [*t*_(654.57)_ = −4.91, *p* < 0.001]. Also, there were different gender distributions found in the Chinese and German samples [China, n_male_>n_female_; Germany, n_male_ < n_female_, χ^2^ = 319.76, df = 1, *p* < 0.001]. Therefore, we further calculated if age and gender influence Internet Literacy, autistic traits and tendencies toward IUD in both samples. For Internet Literacy, we observed significant gender differences in the Chinese sample and the German sample (see Supplementary Tables 2, 3). The associations between age and Internet Literacy dimensions turned out to be different in both samples. In the German sample, age was significantly correlated with all Internet Literacy dimensions, but in the Chinese sample the correlation between age and the dimension *production and interaction* was not significant (see Supplementary Table 4). Regarding autistic traits, the gender effect was significant [*t*_(593)_ = 4.03, *p* < 0.001] and age was significantly correlated with it [*r* = −0.12, *p* = 0.003], but only in the German sample. Regarding tendencies toward IUD, we observed that age was significantly associated with s-IAT scores, but only in the German sample [*r* = −0.22, *p* < 0.001]. For gender effects, male subjects indicated higher s-IAT scores in both samples, but these differences did not reach significance. For an overview of effects of age and gender on the relevant measured variables, please see Supplementary Tables 2–4.

### Correlations Between IUD Tendencies, Internet Literacy, and Autistic Traits

Table 3 shows bivariate correlations with s-IAT scores and ILQ dimension scores as well as the AQ scores, divided by country. To summarize the most important findings: Both in the Chinese and German samples ILQ's *production and interaction* was positively associated with s-IAT scores, whereas ILQ's *self-regulation* was negatively associated with the s-IAT. There was no significant correlation between ILQ's *reflection and critical analysis* and s-IAT scores, neither in the German nor in the Chinese sample. *Technical expertise* significantly correlated with (some) s-IAT scores only in the German sample, and the direction of relationships was mostly divergent in the Chinese and German samples. Fisher's z tests indicated significantly different correlation strength for the s-IAT/ILQ's *technical expertise*, for the s-IAT/ ILQ's *production and interaction* and for the s-IAT/ILQ's *self-regulation* associations between the Chinese and the German sample (see Table 3).

**Table 3:** Correlations between s-IAT scores, AQ scores and ILQ dimension scores (Pearson correlations) including Fisher's z comparison.

	**China (*n* = 929)**			**Germany (*n* = 595)**			**Fisher's** ***z***		
**Domain/Variable**	**s-IAT Total**	**s-IAT LoC/TM**	**s-IAT C/SP**	**s-IAT Total**	**s-IAT LoC/TM**	**s-IAT C/SP**	**s-IAT Total**	**s-IAT LoC/TM**	**s-IAT C/SP**
ILQ TE	−0.004	−0.032	0.026	0.113^**^	0.073	0.140^***^	−2.23^*^	−2.00^*^	−2.18^*^
ILQ PI	0.184^***^	0.146^***^	0.196^***^	0.348^***^	0.251^***^	0.400^***^	−3.36^***^	−2.08^*^	−4.28^***^
ILQ RCA	−0.025	−0.058	0.013	−0.005	−0.010	0.002	−0.38	−0.91	0.21
ILQ SR	−0.297^***^	−0.366^***^	−0.183^***^	−0.536^***^	−0.522^***^	−0.430^***^	5.55^***^	3.71^***^	5.22^***^
AQ Total scores	0.192^***^	0.129^***^	0.229^***^	0.355^***^	0.222^***^	0.452^***^	−3.36^**^	−1.83	−4.83^***^
AQ: High scoring group (CH = 239/G = 126)	−0.121	−0.077	−0.147^*^	0.361^***^	0.304^***^	0.364^***^	−4.49^***^	−3.52^***^	−4.76^**^
AQ: Middle scoring group (CH = 457/G = 337)	0.151^**^	0.103^*^	0.179^***^	0.219^***^	0.150^**^	0.264^***^	−0.98	−0.66	−1.24
AQ: Low scoring group (CH = 233/G = 132)	0.067	0.004	0.130^*^	0.153	0.155	0.111	−0.79	−1.38^**^	0.18

*TE, technical expertise; PI, production and interaction; RCA, reflection and critical analysis; SR, self-regulation; Loc/TM, loss of control/time management; C/SP, craving/social problems; CH, China; G, Germany*;

* *p < 0.05*,

** *p < 0.01*,

*** *p < 0.001*.

The positive associations of AQ total scores with s-IAT scores were found in both countries. Moreover, the correlation between total AQ scores and s-IAT scores was weaker in the Chinese sample compared to the German sample. We also ran detailed analysis for the AQ/s-IAT correlations in the lower, middle and higher AQ scoring groups. Here it became apparent, that the correlation strength between AQ scores and s-IAT scores did not significantly differ in the lower AQ scoring groups between the Chinese sample and the German sample, except the correlation between AQ scores and s-IAT's *loss of control* dimension. In the higher AQ score group, interestingly we see opposing correlation patterns (positive associations in Germany and negative associations in China), which might explain why in the complete sample the positive AQ/s-IAT correlations are weaker in the Chinese compared to the German sample. As can be seen in Table 2 the Chinese and German high AQ scoring groups also differed in terms of the means in each group—the Chinese sample had a significantly higher score. Therefore, it is imaginable that inverse associations between s-IAT scores and AQ scores might also appear in Germany, but only, when much higher AQ scores would be reached (such as in the present Chinese sample). Crossing a certain threshold of AQ scores then might result in contrary correlation patterns (regarding the association with the s-IAT), but such observations need to be backed up by more empirical search and we do not want to over-interpret our findings in this work. Therefore, we will focus in the discussion on the robust positive associations between s-IAT scores and AQ scores observed independently in the complete Chinese and German samples.

For reasons of completeness, we also computed the correlations between ILQ dimensions and AQ scores. Table 4 shows the results from these analysis. In the German sample, except ILQ's *reflection and critical analysis*, all other ILQ dimensions correlated significantly with the AQ scores. Positive associations could be observed between both ILQ's *technical expertise*/*production and interaction* and AQ scores, whereas a negative association could be observed between ILQ's *self-regulation* and AQ scores, similar to the relationship with the s-IAT. In the Chinese sample, we only found correlations in the same directions as found in the German sample for the dimensions of *production and interaction* and *self-regulation*, but the latter did not reach significance (and it was rather a null correlation, although negative). The other two dimensions, *technical expertise/reflection and critical analysis* correlated negatively with AQ scores in the Chinese sample. Fisher's z comparisons revealed that associations were significantly different in the Chinese compared to the German sample. We also compared the correlations between ILQ dimensions and the AQ scores in the lower, middle and higher AQ scoring groups. In the Chinese sample, we did not found any significant correlation in the lower, middle or higher AQ scoring groups. In the German sample, the correlation with ILQ's *production and interaction* was significant in the high AQ scoring group, and the correlations with ILQ *technical expertise/ production and interaction* were significant in the middle AQ scoring group.

**Table 4:** Correlations between Internet Literacy and autistic traits (Pearson correlations) including Fisher's z comparison.

	**China (*n* = 929)**				**Germany (*n* = 595)**				**Fisher's** ***z***			
**Domain/variable**	**ILQ** **TE**	**ILQ** **PI**	**ILQ** **RCA**	**ILQ** **SR**	**ILQ** **TE**	**ILQ** **PI**	**ILQ** **RCA**	**ILQ** **SR**	**ILQ** **TE**	**ILQ** **PI**	**ILQ** **RCA**	**ILQ** **SR**
AQ Total scores	−0.074^*^	0.130^***^	−0.075^*^	−0.024	0.196^***^	0.332^***^	0.034	−0.215^***^	−5.18^***^	−4.07^***^	−2.07^*^	3.69^***^
AQ: High scoring group (CH = 239/G = 126)	−0.081	−0.029	−0.125	−0.059	0.153	0.291^**^	0.022	−0.130	−2.12^*^	−2.96^**^	−1.33	0.64
AQ: Middle scoring group (CH = 457/G = 337)	−0.055	0.004	−0.074	0.019	0.180^***^	0.250^***^	0.034	−0.051	−3.35^***^	−3.49^***^	−1.61	0.92
AQ: Low scoring group (CH = 233/G = 132)	0.046	0.115	0.014	0.020	−0.053	0.062	0.006	−0.086	0.89	0.49	0.05	0.96

*TE, technical expertise; PI, production and interaction; RCA, reflection and critical analysis; SR, self-regulation, CH, China; G, Germany*;

* *p < 0.05*,

** *p < 0.01*,

*** *p < 0.001*.

### Regression Analyses to Predict s-IAT Scores

We report the results of hierarchical regression analysis to predict s-IAT scores in Table 5 for Chinese and German samples. As mentioned before, the gender effect on s-IAT scores was not significant—neither in the Chinese sample nor in the German sample. Therefore, age, AQ scores as well as ILQ dimensions were inserted as independent variables to predict s-IAT scores separately in the Chinese sample and in the German sample.

**Table 5:** Results of hierarchical regression analysis for variables predicting the s-IAT in China and Germany.

	**China (*n* = 929)**				**Germany (*n* = 595)**			
**Predictors**	**B**	**β**	***R*^**2**^ change**	**F change**	**B**	**β**	***R*^**2**^ change**	**F change**
**Model 1**			0.00	0.97			0.05	30.04^***^
Age	−0.26	−0.03			−1.55	−0.22^***^		
**Model 2**			0.04	35.47^***^			0.11	76.76^***^
Age	−0.25	−0.03			−1.26	−0.18^***^		
AQ	1.55	0.19^***^			2.34	0.33^***^		
**Model 3**			0.14	38.00^***^			0.25	62.11^***^
Age	−0.00	0.00			−0.76	−0.11^***^		
AQ	1.37	0.17^***^			1.21	0.17^***^		
ILQ TE	0.37	0.05			−0.18	−0.03		
ILQ PI	1.43	0.18^***^			1.61	0.23^***^		
ILQ RCA	0.57	0.07			−0.04	−0.01		
ILQ SR	−3.02	−0.37^***^			−3.23	−0.46^***^		

*TE, technical expertise; PI, production and interaction; RCA, reflection and critical analysis; SR, self-regulation*;

*** *p < 0.001*.

*All independent variables (e.g., age, AQ scores, ILQ dimensions) were inserted in the model after z-standardization*.

Comparing the results between German and Chinese samples, we found that age as a predictor could explain 5% of the variance in the s-IAT in the German sample, indicating that younger participants in the German sample were more likely to report more severe symptoms of IUD (model 1). Autistic traits were positively related to IUD in both the Chinese and German samples. In detail this model 2 could explain an increment of variance in the s-IAT (China: 4%, Germany: 11%). As for the influence of Internet Literacy, *production and interaction* was significantly positively related to IUD, and *self-regulation* was significantly negatively related to IUD both in the Chinese and German sample. The model 3 including Internet Literacy accounted for an additional 25% of the variance in the s-IAT in the German sample and 14% in the Chinese sample. The overall models both successfully explained the variance in IUD. More details can be found in the Table 5.

### The Interaction Effects Between Internet Literacy and Nationality on IUD Tendencies

Based on the relationships observed between certain Internet Literacy facets/domains and s-IAT scores in both samples, we also calculated further moderated regression analyses to revisit Stodt et al.'s findings (for instance, if possible interaction effects between Internet Literacy and subjects' cultural background could predict individual differences in IUD tendencies) (35).

**Figure 1:**
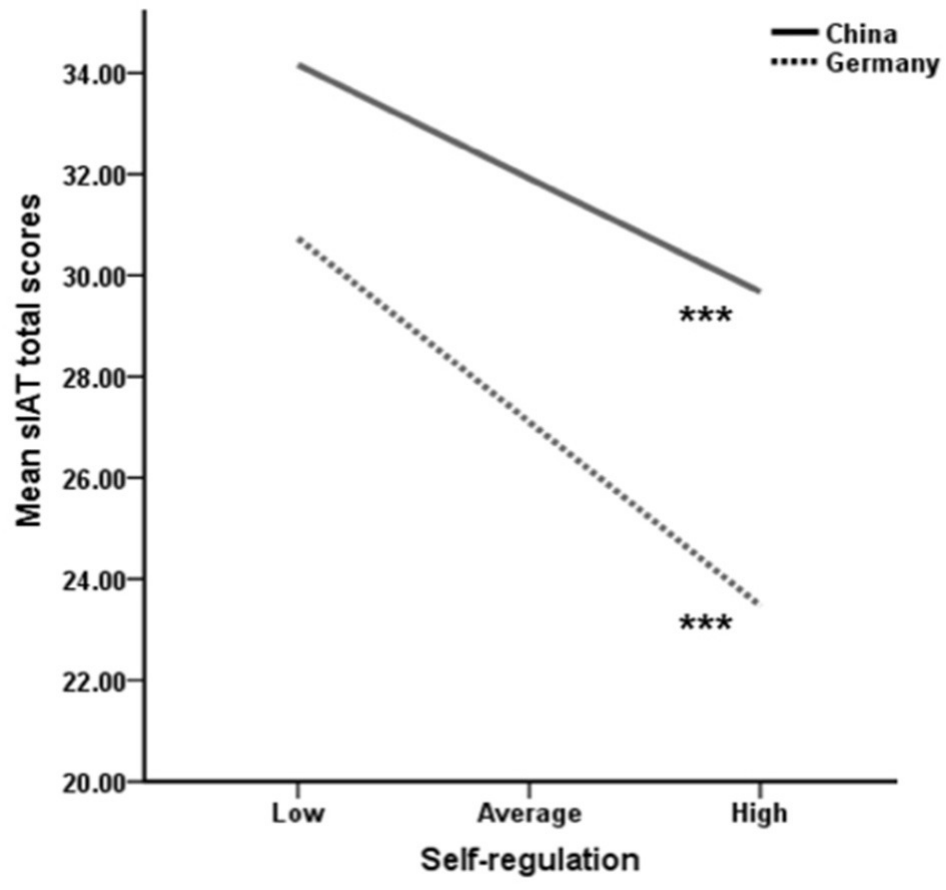
Simple slopes to illustrate the interaction effect between *self-regulation* and country on s-IAT total scores. Note: *** indicates that the s-IAT change from low to high *self-regulation* was significant (*p* < 0.001).

Table 6 presents the moderation effects. As can be seen, after correction for multiple testing (Bonferroni adjustment for four interaction effects to alpha 0.05/4 = 0.0125) the only meaningful interaction is found for the ILQ's *self-regulation* dimension by country on the s-IAT. For visualization of the results, the moderator variable (*self-regulation*) was additionally split into three levels (see Figure 1: low, average and high *self-regulation*). The main effect of *self-regulation* on the s-IAT is similar in both the Chinese and German samples (with lower *self-regulation* going along with higher s-IAT scores), but the association appears to be stronger in the German sample. This finding is also depicted in Figure 1.

**Table 6:** Regression coefficients of the moderated regression analyses with s-IAT total scores as dependent variable.

	**Domain/Variable**	***B***	***SE***	***β***	***t***	***p***
Model 1	ILQ TE	−0.03	0.25	−0.00	−0.11	0.910
	Country	−6.32	0.40	−0.37	−15.66	< 0.001
	Interaction	0.82	0.40	0.06	2.04	0.042
Model 2	ILQ PI	1.49	0.24	0.18	6.08	< 0.001
	Country	−6.32	0.39	−0.37	−16.14	< 0.001
	Interaction	0.96	0.39	0.07	2.46	0.014
Model 3	ILQ RCA	−0.20	0.25	−0.02	−0.79	0.428
	Country	−6.32	0.40	−0.37	−15.63	< 0.001
	Interaction	0.16	0.40	0.01	0.40	0.689
Model 4	ILQ SR	−2.41	0.23	−0.29	−10.34	< 0.001
	Country	−6.32	0.37	−0.37	−16.99	< 0.001
	Interaction	−1.37	0.37	−0.10	−3.68	< 0.001
Model 5	AQ	1.56	0.24	0.19	6.36	< 0.001
	Country	−6.32	0.39	−0.37	−16.17	< 0.001
	Interaction	0.94	0.39	0.07	2.41	0.016

*TE, technical expertise; PI, production and interaction; RCA, reflection and critical analysis; SR, self-regulation*.

*All independent variables (e.g., country, AQ scores, ILQ dimensions) were inserted in the model after z-standardization*.

For reasons of completeness we also report the following: The hierarchical regression with the ILQ's *technical expertise* entered in a first block, country in a second block and the interaction term in a third block explained 14.2% of the variance [Model 1, *F*(3, 1, 520) = 83.86, *p* < 0.001]. The same procedure with the ILQ's *production and interaction* resulted in 19.2% of explained variance [Model 2, *F*(3, 1, 520) = 120.53, *p* < 0.001]. Model 3 with a focus on ILQ's *reflection and critical analysis* revealed 13.9% of explained variance [*F*(3, 1, 520) = 81.66, *p* < 0.001] and Model 4 with a focus on ILQ's *self-regulation* 27.1% of explained variance [*F*(3, 1, 520) = 188.03, *p* < 0.001].

### The Interaction Effects Between the Autistic Traits and Nationality on IUD Tendencies

**Figure 2:**
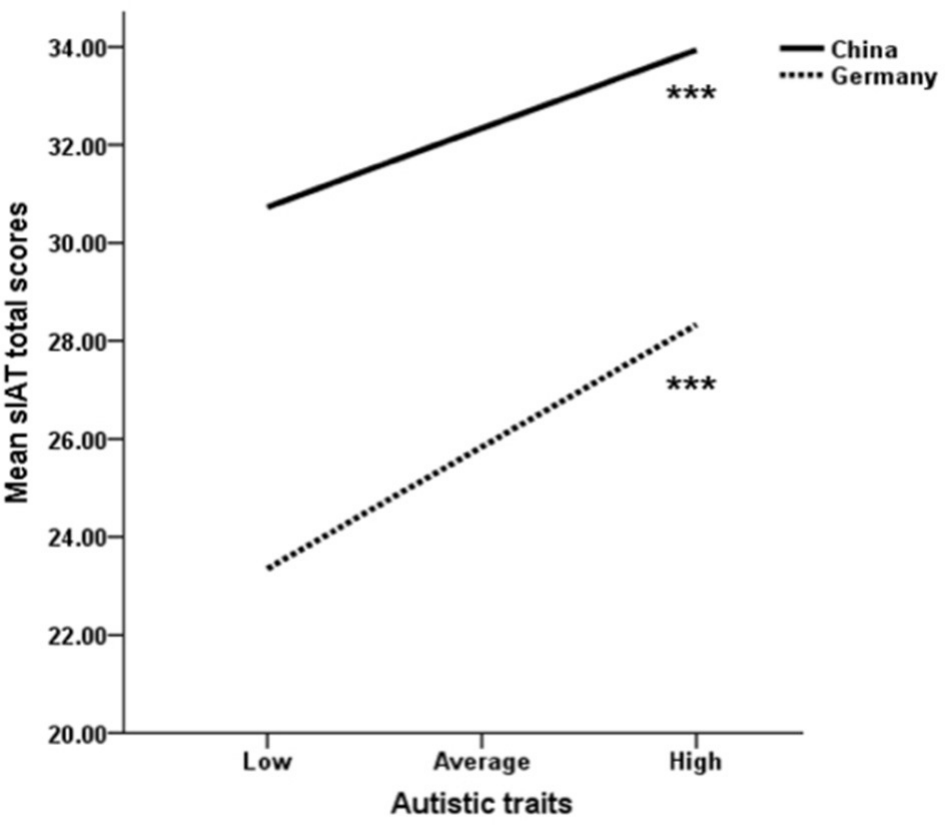
Simple slopes to illustrate the interaction effect between autistic traits and country on the s-IAT total scores. Note: *** indicates that the s-IAT change from low to high autistic traits was significant (*p* < 0.001).

Following the same procedure, we were also interested to examine an interaction effect between the AQ score and country on individual differences in s-IAT scores. As can be seen in Table 6 (Model 5) both the main effect of the AQ on the s-IAT and the main effect of country on the s-IAT were significant. Moreover, the interaction term country x AQ also was significant. In more detail and as depicted in Figure 2, AQ scores predict higher s-IAT scores in both countries, but the simple slope is steeper in the German sample, which is in line with the higher correlations found in the German compared to the Chinese sample (see Figure 2). The overall model 5 explained 19.5% of the variance [*F*(3, 1, 520) = 122.92 *p* < 0.001].

### The Interaction Effects Between Internet Literacy and Autistic Traits on IUD Tendencies in the Chinese and German Samples (Presented Independently)

The relationships between the AQ and the s-IAT, as well as ILQ dimensions and the s-IAT were often significant at the bivariate level. For a deeper understanding of relationships between autistic traits, Internet Literacy, and IUD tendencies, we investigated whether Internet Literacy might moderate the association between autistic traits and IUD tendencies. As the previous hierarchical regression analysis indicated that *production and interaction* and *self-regulatio*n were significantly related to the s-IAT, and age was a significant predictor for the s-IAT in the German sample. We tested the moderated effects with these two dimensions and age, separately in the Chinese and German samples. In block 1, the independent variable, AQ was entered into the model. In block 2, the potential moderators, age, *production and interaction* and *self-regulatio*n were entered into the model. In block 3, the interaction terms with AQ scores were added to the model. Detailed statistical results are presented in Table 7. A summary of model 3: The age effect on the s-IAT was only presented in the Germany sample (β = −0.12, *p* = 0.001), the interaction of AQ × age was not significant, neither in the Chinese nor German sample. In the Chinese sample, the interaction of AQ × *self-regulation* was significant (β = 0.06, *p* = 0.038) whereas in the German sample, the interaction of AQ × *production and interaction* was significant (β = 0.08, *p* = 0.011). Beyond that robust associations between the AQ and s-IAT scores, but also between ILQ's *production and interaction*/ILQ's *self-regulation* scores and s-IAT scores appeared in both China and Germany.

**Table 7:** Regression coefficients of the moderated regression analyses with s-IAT total scores as a dependent variable, AQ total scores, age and ILQ dimensions as independent variables.

**Domain/Variable**	**China (*n* = 929)**				**Germany (*n* = 595)**			
	**B**	**β**	***R*^**2**^ change**	**F change**	**B**	**β**	***R*^**2**^ change**	**F change**
**Model 1**			0.04	35.53^***^			0.13	85.36^***^
AQ	1.56	0.19^***^			2.50	0.35^***^		
**Model 2**			0.13	48.41^***^			0.28	93.27^***^
AQ	1.26	0.16^***^			1.20	0.17^***^		
Age	0.05	0.01			−0.75	−0.11^**^		
ILQ PI	1.76	0.22^***^			1.55	0.22^***^		
ILQ SR	−2.67	−0.33^***^			−3.24	−0.46^***^		
**Model 3**			0.01	2.08			0.01	3.21^*^
AQ	1.24	0.15^***^			1.01	0.14^***^		
Age	0.02	0.00			−0.84	−0.12^**^		
ILQ PI	1.75	0.22^***^			1.49	0.21^***^		
ILQ SR	−2.67	−0.33^***^			−3.22	−0.46^***^		
AQ × age	0.23	0.03			−0.05	−0.01		
AQ × ILQ PI	−0.30	−0.04			0.53	0.08^*^		
AQ × ILQ SR	0.52	0.06^*^			−0.32	−0.05		

*PI, production and interaction; SR, self-regulation*;

* *p < 0.05*;

** *p < 0.01*;

*** *p < 0.001*.

*All independent variables (e.g., age, AQ scores, ILQ PI/SR) were inserted in the model after z-standardization*.

In order to give insights into associations between facets of the AQ and s-IAT scores, we provide correlation patterns in the Supplementary Table 5. Moreover, we present in Table 8 a regression model conducted in the same fashion as the regression model in Table 7 with the exception that in Table 8 the facets of the AQ have been included instead of AQ total scores.

**Table 8:** Regression coefficients of the moderated regression analyses with s-IAT total scores as dependent variable, AQ subscales' scores and ILQ dimensions as independent variables.

**Domain/Variable**	**China (*n* = 929)**				**Germany (*n* = 595)**			
	**B**	**β**	***R*^**2**^ change**	**F change**	**B**	**β**	***R*^**2**^ change**	**F change**
**Model 1**			0.05	9.47^***^			0.15	21.34^***^
AQ SS	0.37	0.05			0.58	0.08		
AQ AS	0.60	0.07^*^			0.86	0.12^**^		
AQ AD	−0.03	−0.00			0.11	0.02		
AQ CO	1.30	0.16^***^			1.85	0.26^***^		
AQ IM	0.08	0.01			0.26	0.04		
**Model 2**			0.13	71.63^***^			0.25	124.83^***^
AQ SS	−0.04	−0.00			−0.05	−0.01		
AQ AS	0.22	0.03			0.85	0.12^***^		
AQ AD	0.17	0.02			0.21	0.03		
AQ CO	1.30	0.16^***^			0.86	0.12^**^		
AQ IM	0.33	0.04			0.10	0.01		
ILQ PI	1.74	0.21^***^			1.64	0.23^***^		
ILQ SR	−2.70	−0.33^***^			−3.29	−0.47^***^		
**Model 3**			0.02	2.53^**^			0.02	1.63
AQ SS	−0.02	−0.00			−0.36	−0.05		
AQ AS	0.27	0.03			0.94	0.13^***^		
AQ AD	0.13	0.02			0.22	0.03		
AQ CO	1.28	0.16^***^			0.73	0.10^*^		
AQ IM	0.27	0.03			0.07	0.01		
ILQ PI	1.72	0.21^***^			1.62	0.23^***^		
ILQ SR	−2.68	−0.33^***^			−3.31	−0.47^***^		
AQ SS × ILQ PI	−0.92	−0.12^**^			0.60	0.10^*^		
AQ SS × ILQ SR	0.18	0.02			−0.11	−0.02		
AQ AS × ILQ PI	0.31	0.04			−0.23	−0.03		
AQ AS × ILQ SR	0.07	0.01			−0.17	−0.02		
AQ AD × ILQ PI	−0.63	−0.08^*^			0.08	0.01		
AQ AD × ILQ SR	−0.28	−0.03			0.02	0.00		
AQ CO × ILQ PI	0.71	0.09^*^			0.05	0.01		
AQ CO × ILQ SR	0.32	0.04			−0.31	−0.05		
AQ IM × ILQ PI	−0.20	−0.03			0.27	0.04		
AQ IM × ILQ SR	0.22	0.03			0.07	0.01		

*AQ facets: SS, social skills; AS, attention switching; AD, attention to details; CO, communication; IM, imagination; ILQ facets: PI, production and interaction, SR, self-regulation*;

* *p < 0.05*;

** *p < 0.01*;

*** *p < 0.001*.

*All independent variables (e.g., AQ facets, ILQ PI/SR) were inserted in the model after z-standardization*.

**Figure 3:**
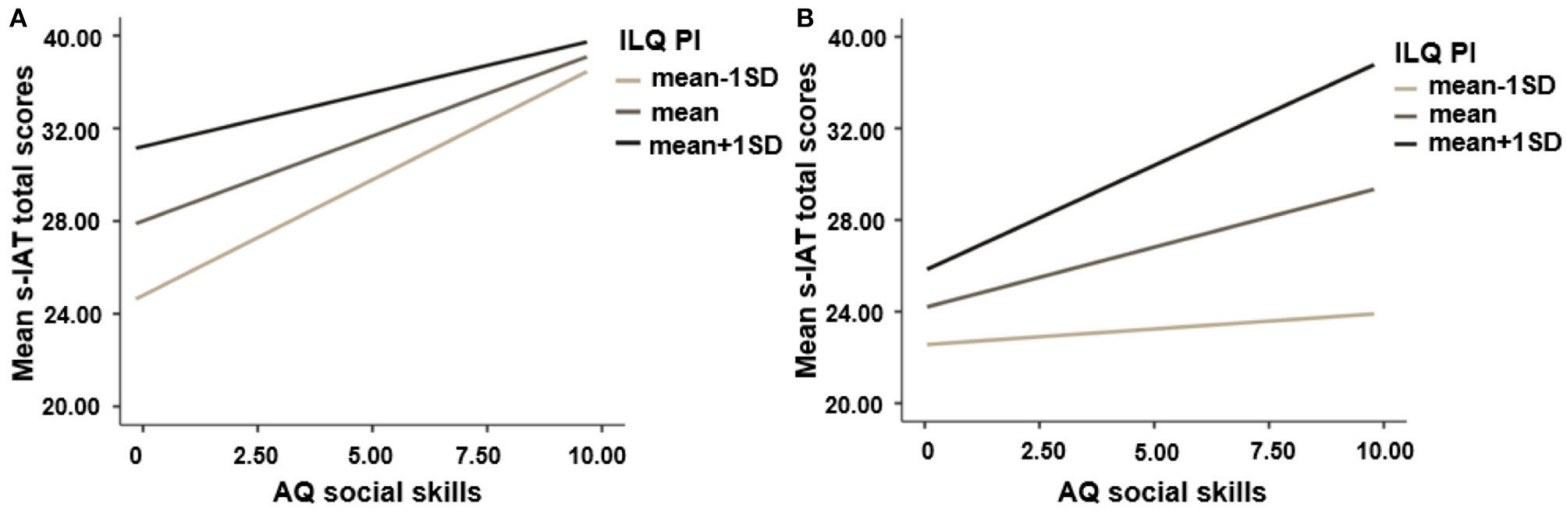
Simple slopes to illustrate the interaction effect between social skills of autistic traits and Internet Literacy *production and interaction* dimension on s-IAT total scores. **(A)** Chinese sample; **(B)** German sample.

As can be seen in Table 8 in model 3 in particular the AQ facet called *communication* predicts s-IAT scores both in the Chinese sample and German sample. Beyond that in both countries a robust interaction between AQ's *social skills* and ILQ's *production and interaction* on s-IAT scores was visible (China, β = −0.12, *p* = 0.002; Germany, β = 0.10, *p* = 0.034; see Figure 3).

## Discussion

This study's goal was to further investigate individual differences in tendencies toward unspecific IUD, and their link to Internet Literacy and autistic traits in two large samples stemming from different cultural backgrounds (China and Germany). Before discussing the results in light of our proposed hypothesis, we should emphasize that in line with previous literature, we observed that IUD levels differed significantly between the Chinese and German samples (26). Against the background of s-IAT scores (assessing IUD) 22.4% of the Chinese sample reported pathological use of the Internet, whereas only 5.5% of the German sample did so. This result is in line with former studies reporting that the prevalence of IUD is higher in Eastern than Western civilizations (rough estimate) and might reflect cultural differences (49). Cultural differences were also observed for the Internet Literacy variable. The current results indicated higher *technical expertise*, higher *production and interaction*, higher *reflection and critical analysis* as well less *self-regulation* skills in the Chinese sample compared to the German sample. These results were in line with observations of a previous study except the dimension *reflection and critical analysis*. In the earlier work by Stodt et al. (35) in the German sample higher scores on this scale compared to the Chinese sample were reported. Regarding higher scores in the ILQ variables *technical expertise/production and interaction* in the Chinese compared to the German sample we think this might reflect a keen focus in Chinese education on technical skills, but this idea needs further empirical backup.

In general, the results of the present work confirm earlier observations that higher autistic traits are linked to higher tendencies toward IUD (5, 31). In the present study, positive correlations between autistic traits and IUD tendencies were found in both the Chinese and German sample, with correlations of 0.19 (China) and 0.36 (Germany), indicating robust although low to medium effect sizes. The analysis of AQ facets revealed that higher communication problems are in particular related to higher IUD tendencies (see Supplementary Table 5). As this is (according to our best knowledge) the first study also reporting associations between AQ's facets and s-IAT scores, these findings need to be replicated and should be seen as preliminary. We earlier mentioned the exploratory character of our study, here.

Stodt et al. (35) previously also investigated the association between Internet Literacy and IUD in independent samples of both Chinese and German subjects using comparable methods as in the present work regarding the assessment of these variables (administration of the ILQ's self-report and the s-IAT measures). They observed in both their Chinese and German samples that ILQ's *technical expertise*/*production and interaction* were positively linked to IUD tendencies. Therefore, we also wanted to revisit these findings again. In the present study again positive association between ILQ's *technical expertise* and IUD tendencies could be observed in the German sample while this association was close to zero in China. In the present study again positive associations between ILQ's *production and interaction* and IUD tendencies could be observed in both countries. Hence, this finding is very robust. High scores on this facet describe individuals who state that the Internet is a good place to make contacts and to interact with others, even preferable to the offline world. Individuals with high ILQ's *production and interaction* also mention that they find it easier to be creative online and formulate an opinion online compared to the offline world. It is very straightforward to propose that high scores on this facet would go along with higher autistic traits, although we did not set up such a hypothesis. Indeed, results from Supplementary Table 6 supports this notion with positive associations between ILQ's *production and interaction* and AQ scores both in China and Germany. Furthermore, our moderated analyses at least for the German sample provide support for the idea that higher autistic traits might lead to a greater preference for online interactions (hence *production and interaction*) and thereby result in a greater risk of developing IUD. We also mention that the current study showed significant differences in the strength of effects sizes regarding all Internet Literacy dimensions and IUD tendencies associations between the Chinese and German samples (lower in China compared to Germany), except the association between ILQ's *reflection and critical analysis*. Beyond these insights, it needs to be mentioned that ILQ's *self-regulation* was negatively associated with IUD in China and Germany.

The current study has some limitations, which should be mentioned. First of all, the present work relied on self-report questionnaires and answers given may be influenced by tendencies to answer in a socially desirable manner. Second, we did not investigate clinical samples in China and Germany and findings might differ in individuals diagnosed with autism, even though the dimensional approach to understand psychopathological phenotypes is well-established in a range of psychopathological conditions. Third, samples recruited in China and Germany differ in some socio-demographic variables. Hence it is not clear whether differences in both samples are due to culture or other variables. Socio-demographic variables could have been also assessed beyond age, gender and education, but our study is limited by a focus on rather young participants (mostly student background). Next, comorbid other relevant psychiatric symptoms such as depressive tendencies (but see some analysis in the Supplementary Material (part 2) regarding depressive tendencies) have not been taken into account in the present work. Finally, our findings are only of correlational nature and no specific causality can be derived. We also want to mention recent discussions about the best way to analyze facets of the AQ: English et al. (50) provided evidence to analyze the AQ with a three factor solution. Of note, as the present work in particular focused on the AQ facets *social skills* and *communication*—and we here observed reasonable internal consistencies—we stuck with our analysis method (taking into account five facets although some of them showed weak psychometrics). But again, a three factor solution as proposed by English et al. (50) with the factors *social skill, patterns/details* and *communication/mindreading* would be also interesting. Finally, we mentioned earlier that the ILQ findings should be seen as of preliminary nature.

In summary, we have provided further support for an association between higher autistic traits and higher tendencies toward IUD. As demonstrated from our moderation analysis, this association might be explained by autistic individuals favoring online vs. offline social interactions providing them with a more secure and comfortable environment in which to communicate (at least this is supported by the German data). A negative side effect of such an adaptation could however be the development of unhealthy online behaviors.

## Conclusion

The present study revisited previous findings on IUD tendencies and autistic traits (5, 31), additionally taking into account interaction effects between ILQ domains and autistic traits in China and Germany. The association between IUD tendencies and autistic traits in parts were moderated by facets of Internet Literacy.

The prevalence of IUD was higher in the Chinese compared to the German sample, which is in line with previous studies. In addition, higher autistic traits were linked to higher tendencies toward IUD in both the Chinese and German samples and this link is moderated by Internet Literacy domains such as *production and interaction* and *self-regulation* (with different observations in China and Germany). In so far, the present study provides evidence that cultural differences may also play a role in the development and maintenance of IUD. Against the background of the correlational nature of the present study, next steps would be to run longitudinal studies to get insights into causality between variables such as IUD tendencies and autistic traits. Such an approach would be interesting to be conducted also in samples with different cultural background (such as from China and Germany) to investigate if same causality applies perhaps even independent of cultural aspects.

## Data Availability Statement

The raw data supporting the conclusions of this article will be made available by the authors, upon scholarly request.

## Ethics Statement

The studies involving human participants were reviewed and approved by the ethic committees at UESTC and Ulm University. The patients/participants provided their written informed consent to participate in this study.

## Author Contributions

CM and YZ designed the present study. YZ wrote the first version of the manuscript and conducted the statistical analysis. CS double checked the statistical analysis. CM, BB, KMK, and CS critically revised the manuscript and approved the final version of the manuscript for submission. All authors contributed to the article and approved the submitted version.

## Conflict of Interest

The authors declare that the research was conducted in the absence of any commercial or financial relationships that could be construed as a potential conflict of interest.

## Publisher's Note

All claims expressed in this article are solely those of the authors and do not necessarily represent those of their affiliated organizations, or those of the publisher, the editors and the reviewers. Any product that may be evaluated in this article, or claim that may be made by its manufacturer, is not guaranteed or endorsed by the publisher.

## Footnotes

^1^https://www.internetworldstats.com/stats.htm (accessed July 22, 2021).

^2^https://icd.who.int/browse11/l-m/en#/http://id.who.int/icd/entity/1448597234 (accessed May 11, 2021).
