# Supplementary material for: Individual Differences in Tendencies Toward Internet Use Disorder, Internet Literacy and Their Link to Autistic Traits in Both China and Germany
Source: Front Psychiatry. 2021 Sep 27;12:638655. doi: 10.3389/fpsyt.2021.638655 (PMC8502933; doi:10.3389/fpsyt.2021.638655)
Supplement: Supplementary file 1 [file Data_Sheet_1.docx]

**Supplementary Material**

# Individual differences in tendencies towards Internet Use Disorder, Internet Literacy and their link to autistic traits in both China and Germany

# YingYing Zhang^1^, Cornelia Sindermann^1^, Keith M. Kendrick^2^, Benjamin Becker^2^ & Christian Montag^1,2*^

^1^Department of Molecular Psychology, Institute of Psychology and Education, Ulm University, Ulm, Germany

^2^The Clinical Hospital of Chengdu Brain Science Institute, MOE Key Lab for Neuroinformation, University of Electronic Science and Technology of China, Chengdu, China

**Part 1**

**Distributions of s-IAT scores, ILQ dimensions and AQ scores presented separately for the Chinese and German sample**

We tested for normal distribution with skewness and kurtosis (variables with skewness and kurtosis <+/-2 were considered as normally distributed ([Curran, West, & Finch, 1996](#_ENREF_1))). Results of the skewness and kurtosis are depicted in details in **Supplementary Table 1** (separately for China and Germany).

**Supplementary Table 1.** Skewness and Kurtosis of all scales under investigation in the Chinese and German samples.

|  | **s-IAT** | **ILQ TE** | **ILQ PI** | **ILQ RCA** | **ILQ SR** | **AQ** |
| --- | --- | --- | --- | --- | --- | --- |
| **China** |  |  |  |  |  |  |
| Skewness | 0.339  SE=0.080 | -0.348  SE=0.080 | -0.301  SE=0.080 | -0.142  SE=0.080 | -0.100  SE=0.080 | -0.025  SE=0.080 |
| Kurtosis | 0.388  SE=0.160 | -0.116  SE=0.160 | 0.124  SE=0.160 | 0.182  SE=0.160 | 0.137  SE=0.160 | -0.265  SE=0.160 |
| **Germany** |  |  |  |  |  |  |
| Skewness | 0.746  SE=0.100 | 0.086  SE=0.100 | 0.209  SE=0.100 | -0.411  SE=0.100 | -0.395  SE=0.100 | 0.519  SE=0.100 |
| Kurtosis | 0.968  SE=0.200 | -0.645  SE=0.200 | -0.494  SE=0.200 | -0.137  SE=0.200 | -0.103  SE=0.200 | 0.064  SE=0.200 |

TE = technical expertise, PI = production and interaction, RCA = reflection and critical analysis, SR = self-regulation


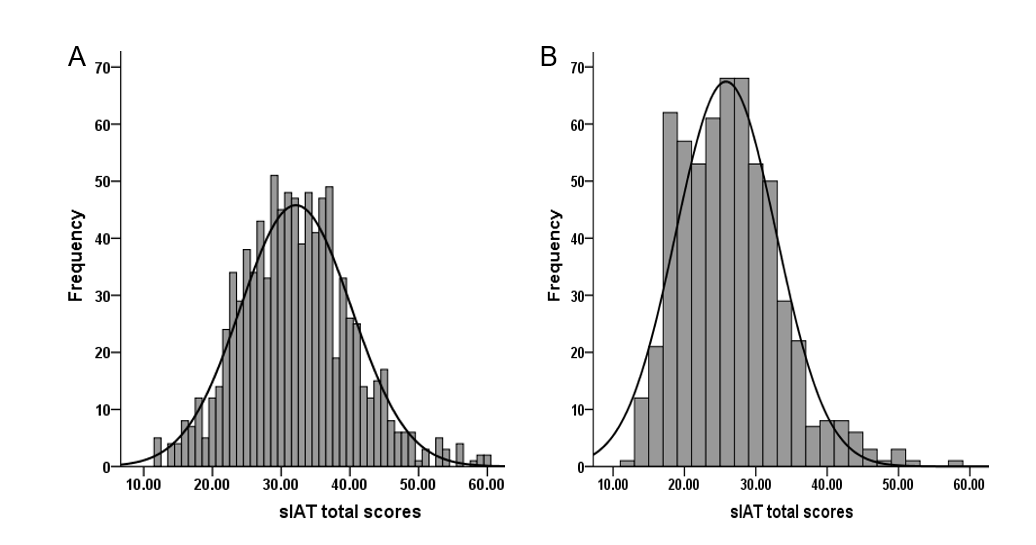

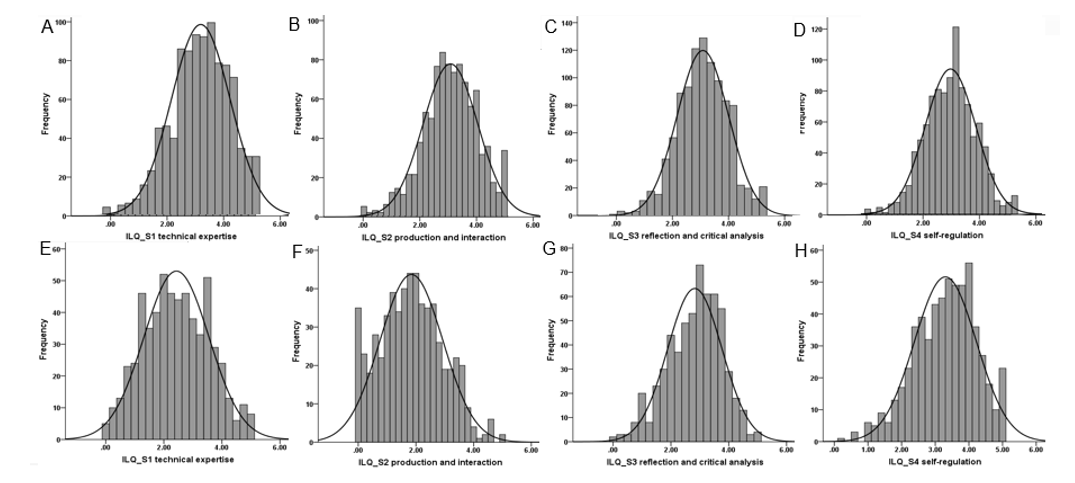
**Supplementary Figure 1**. Histograms of the scores in s-IAT scores (A = Chinese sample; B = German sample).


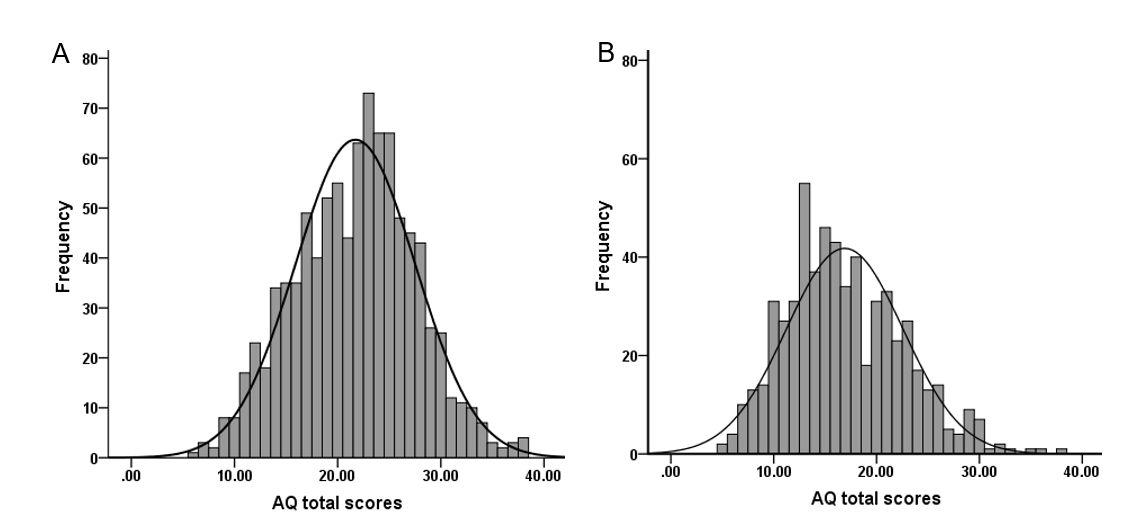
**Supplementary Figure 2**. Histograms of the scores in the ILQ dimensions (A-D: Chinese sample; E-H: German sample).

**Supplementary Figure 3.** Histograms of the scores in AQ scores (A = Chinese sample; B = German sample).

**Supplementary Table** **2.** Gender differences in the s-IAT, ILQ dimensions and AQ scores as measured by means of t-tests in the Chinese sample.

| **Domain/variable** | **China (n = 929)** | | | |  |  |  |
| --- | --- | --- | --- | --- | --- | --- | --- |
|  | **Male (n = 696)** | | **Female (n = 233)** | |  |  |  |
|  | M | SD | M | SD | t | df | p |
| **short Internet Addiction Test (s-IAT)** | 32.28 | 8.13 | 31.72 | 7.97 | 0.92 | 927 | 0.357 |
| **Internet literacy (ILQ)** |  |  |  |  |  |  |  |
| Technical expertise | 3.34 | 0.92 | 2.60 | 0.96 | 10.45 | 927 | <0.001 |
| Production and interaction | 3.14 | 0.94 | 2.89 | 1.04 | 3.32 | 927 | <0.001 |
| Reflection and critical analysis | 3.08 | 0.84 | 2.71 | 0.86 | 5.69 | 927 | <0.001 |
| Self-regulation | 2.87 | 0.85 | 2.66 | 0.88 | 3.21 | 927 | 0.001 |
| **Autistic traits (AQ)** | 21.81 | 5.87 | 21.42 | 5.66 | 0.87 | 927 | 0.384 |

**Supplementary Table** **3.** Gender differences in the s-IAT, ILQ dimensions and AQ scores as measured by means of t-tests in the German sample.

| **Domain/variable** | **Germany (n = 595)** | | | |  |  |  |
| --- | --- | --- | --- | --- | --- | --- | --- |
|  | **Male (n = 169)** | | **Female (n = 426)** | |  |  |  |
|  | M | SD | M | SD | t | df | p |
| **short Internet Addiction Test (s-IAT)** | 26.58 | 7.07 | 25.52 | 7.01 | 1.66 | 593 | 0.097 |
| **Internet literacy (ILQ)** |  |  |  |  |  |  |  |
| Technical expertise | 3.07 | 1.08 | 2.19 | 1.04 | 9.19 | 593 | <0.001 |
| Production and interaction | 2.11 | 1.12 | 1.75 | 1.06 | 3.73 | 593 | <0.001 |
| Reflection and critical analysis | 3.05 | 0.89 | 2.75 | 0.95 | 3.44 | 593 | <0.001 |
| Self-regulation | 2.98 | 0.96 | 3.43 | 0.87 | -5.56 | 593 | <0.001 |
| **Autistic traits (AQ)** | 18.37 | 5.82 | 16.32 | 5.53 | 4.03 | 593 | <0.001 |

**Supplementary Table** **4.** Correlations between age and the variables of interest.

| **Domain/variable** | **Age** | | |
| --- | --- | --- | --- |
|  | **Total**  **(n = 1524)** | **China**  **(n = 929)** | **Germany**  **(n = 595)** |
| **short Internet Addiction Test (s-IAT)** | -0.173^***^ | -0.032 | -0.220^***^ |
| **Internet literacy (ILQ)** |  |  |  |
| Technical expertise | -0.087^***^ | 0.142^***^ | -0.122^**^ |
| Production and interaction | -0.165^***^ | -0.009 | -0.163^***^ |
| Reflection and critical analysis | -0.049 | 0.067^*^ | -0.087^*^ |
| Self-regulation | 0.138^***^ | 0.109^***^ | 0.124^**^ |
| **Autistic traits (AQ)** | -0.123^***^ | -0.005 | -0.121^**^ |

*= *p* <0.05, **= *p* < 0.01, ***= *p* < 0.001

**Supplementary Table 5.** Correlations between the s-IAT and AQ total scores as well as AQ subscale’s scores (Pearson correlations) including Fisher’s z comparison.

|  | **China (n = 929)** | | |  | | **Germany (n= 595)** | | |  |
| --- | --- | --- | --- | --- | --- | --- | --- | --- | --- |
| **Domain**  **/Variable** | **s-IAT**  **Total** | **s-IAT LoC/TM** | **s-IAT**  **C/SP** |  | **s-IAT**  **Total** | | **s-IAT LoC/TM** | **s-IAT**  **C/SP** |  |
| **AQ Total** | 0.192^***^ | 0.129^***^ | 0.229^***^ |  | 0.355^***^ | | 0.222^***^ | 0.452^***^ |  |
| SS | 0.152^***^ | 0.104^**^ | 0.178^***^ |  | 0.274^***^ | | 0.145^***^ | 0.385^***^ |  |
| AS | 0.116^***^ | 0.104^**^ | 0.111^***^ |  | 0.238^***^ | | 0.184^***^ | 0.257^***^ |  |
| AD | -0.028 | -0.033 | -0.019 |  | 0.026 | | -0.001 | 0.058 |  |
| CO | 0.202^***^ | 0.137^***^ | 0.239^***^ |  | 0.360^***^ | | 0.259^***^ | 0.414^***^ |  |
| IM | 0.094^**^ | 0.049 | 0.126^***^ |  | 0.176^***^ | | 0.085^*^ | 0.259^***^ |  |

Loc/TM = loss of control/time management, C/SP = craving/social problems, SS = social skills; AS = attention switching; AD = attention to details; CO = communication; IM = imagination, *= *p* <0.05, **= *p* <0.01, ***= *p* <0.001

**Supplementary Table 6.** Correlations between ILQ dimensions and AQ total scores as well as AQ subscales’ scores (Pearson correlations) including Fisher’s z comparison.

| **Domain/**  **Variable** | **China (n=929)** | | | |  | **Germany(n=595)** | | | |
| --- | --- | --- | --- | --- | --- | --- | --- | --- | --- |
|  | **ILQ**  **TE** | **ILQ**  **PI** | **ILQ**  **R/CA** | **ILQ**  **SR** |  | **ILQ**  **TE** | **ILQ**  **PI** | **ILQ**  **R/CA** | **ILQ**  **SR** |
| **AQ Total** | -0.074^*^ | 0.130^***^ | -0.075^*^ | -0.024 |  | 0.196^***^ | 0.332^***^ | 0.034 | -0.215^***^ |
| SS | -0.150^***^ | 0.055 | -0.176^***^ | -0.126^***^ |  | 0.138^***^ | 0.305^***^ | -0.011 | -0.222^***^ |
| AS | -0.111^***^ | 0.078^*^ | -0.113^***^ | -0.117^***^ |  | 0.009 | 0.169^***^ | -0.040 | -0.080 |
| AD | 0.177^***^ | 0.088^**^ | 0.222^***^ | 0.156^***^ |  | 0.233^***^ | 0.066 | 0.154^***^ | 0.057 |
| CO | -0.100^**^ | 0.111^***^ | -0.100^**^ | -0.006 |  | 0.075 | 0.305^***^ | -0.023 | -0.268^***^ |
| IM | -0.006 | 0.037 | -0.020 | 0.041 |  | 0.127** | 0.153^***^ | 0.014 | -0.154^***^ |

TE = technical expertise, PI = production and interaction, RCA = reflection and critical analysis, SR = self-regulation SS = social skills; AS = attention switching; AD = attention to details; CO = communication; IM = imagination, *= *p* <0.05, **= *p* <0.01, ***= *p* <0.001

**Supplementary Table 7.** Differences in s-IAT scores and ILQ dimensions depending on the different levels of autistic traits in China and Germany.

| **Domain/Variable** | **AQ （cutoff, ≥26）** | | | | | | | | | |
| --- | --- | --- | --- | --- | --- | --- | --- | --- | --- | --- |
| **Domain/**  **Variable** | **China (n=929)**  **t**  **p** | | | | | **Germany (n=595)** | | | | |
|  | **Low AQ**  **(n=690)** | **High AQ**  **(n=239)** |  |  |  | **Low AQ**  **(n=549)** | **High AQ**  **(n=46)** |  |  |  |
|  | **M (SD)** | **M (SD)** | **t** | **df** | **p** | **M (SD)** | **M (SD)** | **t** | **df** | **p** |
| **s-IAT Total** | 31.62 (7.93) | 33.64 (8.37) | -3.34 | 927 | <0.001 | 25.25 (6.68) | 32.61 (7.74) | -7.09 | 593 | <0.001 |
| **s-IAT LoC/TM** | 17.24 (4.39) | 18.01 (4.48) | -2.33 | 927 | 0.020 | 15.41 (4.43) | 18.74 (4.30) | -4.91 | 593 | <0.001 |
| **s-IAT C/SP** | 14.38 (4.18) | 15.63 (4.50) | -3.90 | 927 | <0.001 | 9.85 (3.03) | 13.87 (4.16) | -6.42 | 49.09 | <0.001 |
| **ILQ** | | | | | | | | | | |
| **TE** | 3.15 (0.99)  ( | 3.15 (0.98) | 0.09 | 927 | 0.931 | 2.41 (1.12) | 2.80 (1.06) | -2.28 | 593 | 0.023 |
| **PI** | 3.01 (0.98) | 3.27 (0.94) | -3.57 | 927 | <0.001 | 1.79 (1.06) | 2.56 (1.15) | -4.64 | 593 | <0.001 |
| **R/CA** | 3.00 (0.85) | 2.95 (0.90) | 0.76 | 927 | 0.450 | 2.84 (0.94) | 2.85 (0.98) | -0.08 | 593 | 0.933 |
| **SR** | 2.81 (0.86) | 2.85 (0.86) | -0.64 | 927 | 0.520 | 3.37 (0.90) | 2.59 (0.94) | 5.64 | 593 | <0.001 |
|  | **AQ （cutoff, ≥32）** | | | | | | | | | |
|  | **(n=889)** | **(n=40)** |  |  |  | **(n=589)** | **(n=6)** |  |  |  |
| **s-IAT Total** | 32.15 (8.08) | 31.85 (8.47) | 0.23 | 927 | 0.817 | 25.70 (6.96) | 37.00 (6.26) | -3.96 | 593 | <0.001 |
| **s-IAT LoC/TM** | 17.44 (4.41) | 17.45 (4.71) | -0.02 | 927 | 0.984 | 15.61 (4.49) | 20.67 (3.83) | -2.75 | 593 | 0.006 |
| **s-IAT C/SP** | 14.72 (4.29) | 14.40 (4.56) | 0.46 | 927 | 0.648 | 10.10 (3.25) | 16.33 (3.39) | -4.68 | 593 | <0.001 |
| **ILQ** | | | | | | | | | | |
| **TE** | 3.16 (0.99)  ( | 2.93 (0.93) | 1.44 | 927 | 0.151 | 2.43 (1.12) | 3.29 (0.90) | -1.87 | 593 | 0.062 |
| **PI** | 3.07 (0.98) | 3.11 (0.91) | -0.19 | 927 | 0.848 | 1.84 (1.08) | 3.40 (1.36) | -3.53 | 593 | <0.001 |
| **R/CA** | 3.00 (0.86) | 2.63 (0.80) | 2.73 | 927 | 0.006 | 2.84 (0.94) | 2.92 (1.30) | -0.21 | 593 | 0.834 |
| **SR** | 2.82 (0.86) | 2.74 (0.91) | 0.57 | 927 | 0.569 | 3.31 (0.93) | 3.17 (0.67) | 0.37 | 593 | 0.712 |

Loc/TM = loss of control/time management, C/SP = craving/social problems; TE = technical expertise, PI = production and interaction, RCA = reflection and critical analysis, SR = self-regulation

**Supplementary** **Table 8**. Differences in AQ scores and the AQ subscale-scores depending on the different Internet user groups in China and Germany.

|  | **No**  **problematic**  **Internet use** | **Problematic Internet use** | **Pathological Internet use** | **F** | **df** | **p** |
| --- | --- | --- | --- | --- | --- | --- |
| **China** | (n = 402) | (n = 319) | (n = 208) |  |  |  |
| **AQ Total** | 20.60 (6.23) | 21.92 (5.62) | 23.54 (4.74) | 18.52 | 2,926 | <0.001 |
| SS | 3.52 (2.61) | 4.11 (2.56) | 4.42 (2.21) | 10.14 | 2,926 | <0.001 |
| AS | 4.99 (1.77) | 5.37 (1.66) | 5.44 (1.83) | 6.43 | 2,926 | 0.002 |
| AD | 5.04 (2.15) | 4.81 (2.01) | 4.98 (1.87) | 1.22 | 2,926 | 0.295 |
| CO | 3.30 (2.19) | 3.68 (1.94) | 4.42 (1.85) | 20.86 | 2,926 | <0.001 |
| IM | 3.75 (1.78) | 3.96 (1.81) | 4.29 (1.87) | 6.00 | 2,926 | 0.003 |
| **Germany** | (n=456) | (n=106) | (n=33) |  |  |  |
| **AQ Total** | 16.06 (5.14) | 18.76 (6.09) | 22.55 (6.84) | 29.58 | 2,592 | <0.001 |
| SS | 1.69 (1.81) | 2.56 (2.50) | 3.64 (2.55) | 20.50 | 2,592 | <0.001 |
| AS | 4.77 (1.90) | 5.64 (1.85) | 5.94 (2.16) | 13.35 | 2,592 | <0.001 |
| AD | 5.53 (2.05) | 5.49 (2.15) | 6.06 (1.89) | 1.08 | 2,592 | 0.342 |
| CO | 2.02 (1.68) | 2.82 (2.00) | 4.12 (2.06) | 28.18 | 2,592 | <0.001 |
| IM | 2.05 (1.50) | 2.25 (1.59) | 2.79 (1.63) | 4.04 | 2,592 | 0.018 |

SS = social skills; AS = attention switching; AD = attention to details; CO = communication; IM = imagination

**Part 2**

Aside from presenting the histograms, we aimed to replicate the findings by Romano (2014) in the Chinese sample ([Romano et al., 2014](#_ENREF_2)). Please note that we did not have the STAI measure in the German database.

We used the same cut-off-scores (cut-off of 32 or higher) as Romano (2014) to split the sample into lower and higher autistic traits and lower and higher anxiety groups ([Romano et al., 2014](#_ENREF_2)). **Figure 4** of this supplement showed the scores of the s-IAT depending on 4 groups defined by lower- and higher-anxiety (STAI-T) and two autistic trait (AQ) groups (hence we have a 2 x 2 design investigating s-IAT scores).

**Supplementary Table 9.** Means (standard deviations) for all scales and the Pearson correlations between these scales

| **Scale** | **Mean (SD)** | **(1)** | **(2)** | **(3)** |
| --- | --- | --- | --- | --- |
| **STAI-T(1)** | 41.58 (8.84) |  |  |  |
| **AQ(2)** | 21.80 (5.69) | 0.019 |  |  |
| **BDI(3)** | 28.21 (8.26) | 0.565^***^ | 0.009 |  |
| **s-IAT(4)** | 32.40 (8.06) | 0.116^***^ | 0.203^***^ | 0.064 |

*= *p* <0.05, **= *p* <0.01, ***= *p* <0.001 (two-tailed)

To determine statistical differences between groups with respect to IUD tendencies, an ANCOVA, with anxiety (lower versus higher) and autistic traits (lower versus higher) as factors, and depression (BDI) as a covariate variable, was conducted. This ANCOVA indicated a statistically significant main effect of autistic traits (F_(1, 924)_ = 44.69, *p* < 0.001, partial eta^2^ = 0.046). There was no main effect of anxiety (F_(1, 924)_ = 3.08, *p* =0.080, partial eta^2^ = 0.003). In addition, no statistically significant interaction between the factors of autistic traits/anxiety on IUD could be observed (F_(1, 924)_ = 0.50, p = 0.480，partial eta^2^ <0.001). Please note that in the work by [Romano et al. (2014](#_ENREF_2)) not only the main effect of the AQ on IUD tendencies was significant, but also the main effect of anxiety on IUD tendencies. The latter was not the case in the present work (although a trend was visible). Beyond this, also the low AQ/high anxiety group showed much higher scores in the Romano et al. sample than in the present work (but please note that the interaction effect was also not significant in [Romano et al. (2014](#_ENREF_2))).


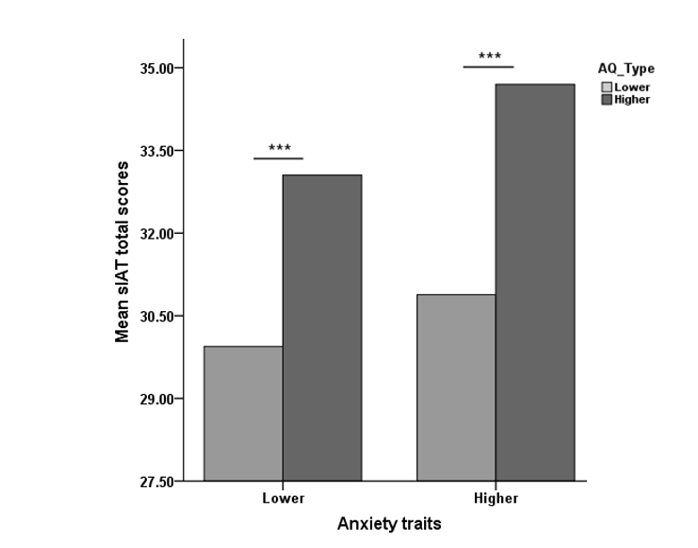
**Supplementary Figure 4.** Means of the s-IAT for four groups defined by a combination of lower and higher autistic traits and lower- and higher anxiety scores

In conclusion, the results from our Chinese sample were in parts consistent with those by [Romano et al. (2014](#_ENREF_2)), confirming the association between autistic traits and IUD tendencies. Beyond this we see also differences, which warrant further investigations.

**References**

Curran, P. J., West, S. G., & Finch, J. F. (1996). The robustness of test statistics to nonnormality and specification error in confirmatory factor analysis. *Psychological methods, 1*(1), 16.

Romano, M., Truzoli, R., Osborne, L. A., & Reed, P. (2014). The relationship between autism quotient, anxiety, and internet addiction. *Research in Autism Spectrum Disorders, 8*(11), 1521-1526.
